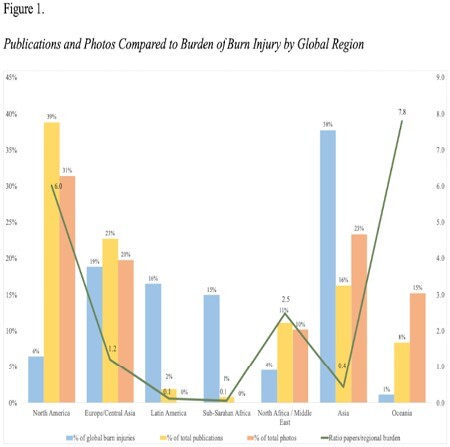# 602 Too Few Burn Papers and Images, Lowering Publication Barriers for Low- and Middle-Income Country Authors

**DOI:** 10.1093/jbcr/irae036.236

**Published:** 2024-04-17

**Authors:** Cameron J Kneib, Ryann E Shor, Kiran K Nakarmi, Manish K Yadav, Raslina Shrestha, Ariel Miranda, Tam N Pham, Barclay T Stewart

**Affiliations:** UW Medicine Regional Burn Center, Seattle, WA; Kirtipur Hospital, Bagmati, Bagmati; Kirtipur Hospital, Kathmandu, Bagmati; Hospital Civil de Guadalajara, Gaudalajara, Jalisco; University of Washington, Harborview Burn Centre, Seattle, WA; University of Washington, Seattle, WA; UW Medicine Regional Burn Center, Seattle, WA; Kirtipur Hospital, Bagmati, Bagmati; Kirtipur Hospital, Kathmandu, Bagmati; Hospital Civil de Guadalajara, Gaudalajara, Jalisco; University of Washington, Harborview Burn Centre, Seattle, WA; University of Washington, Seattle, WA; UW Medicine Regional Burn Center, Seattle, WA; Kirtipur Hospital, Bagmati, Bagmati; Kirtipur Hospital, Kathmandu, Bagmati; Hospital Civil de Guadalajara, Gaudalajara, Jalisco; University of Washington, Harborview Burn Centre, Seattle, WA; University of Washington, Seattle, WA; UW Medicine Regional Burn Center, Seattle, WA; Kirtipur Hospital, Bagmati, Bagmati; Kirtipur Hospital, Kathmandu, Bagmati; Hospital Civil de Guadalajara, Gaudalajara, Jalisco; University of Washington, Harborview Burn Centre, Seattle, WA; University of Washington, Seattle, WA; UW Medicine Regional Burn Center, Seattle, WA; Kirtipur Hospital, Bagmati, Bagmati; Kirtipur Hospital, Kathmandu, Bagmati; Hospital Civil de Guadalajara, Gaudalajara, Jalisco; University of Washington, Harborview Burn Centre, Seattle, WA; University of Washington, Seattle, WA; UW Medicine Regional Burn Center, Seattle, WA; Kirtipur Hospital, Bagmati, Bagmati; Kirtipur Hospital, Kathmandu, Bagmati; Hospital Civil de Guadalajara, Gaudalajara, Jalisco; University of Washington, Harborview Burn Centre, Seattle, WA; University of Washington, Seattle, WA; UW Medicine Regional Burn Center, Seattle, WA; Kirtipur Hospital, Bagmati, Bagmati; Kirtipur Hospital, Kathmandu, Bagmati; Hospital Civil de Guadalajara, Gaudalajara, Jalisco; University of Washington, Harborview Burn Centre, Seattle, WA; University of Washington, Seattle, WA; UW Medicine Regional Burn Center, Seattle, WA; Kirtipur Hospital, Bagmati, Bagmati; Kirtipur Hospital, Kathmandu, Bagmati; Hospital Civil de Guadalajara, Gaudalajara, Jalisco; University of Washington, Harborview Burn Centre, Seattle, WA; University of Washington, Seattle, WA

## Abstract

**Introduction:**

Whereas the burden of burn injury is greatest in low- and middle-income countries (LMIC), publications from these regions represent a disproportionately small volume of the published burn literature in English. This important gap in our cumulative knowledge contributes to the slow progress in global burn injury control. In addition to manuscripts, clinical photographs are important representations of injury and outcomes. We aimed to compare the current volume of burn publications and photographs to the global burn injury burden and sought to identify potential journal-level barriers to publication.

**Methods:**

A bibliometric analysis was performed by sampling three leading burn surgery journals published in the English language (Journal of Burn Care and Research, Burns, European Burn Journal) between 2021-2023. Country of corresponding author and number of photos were tabulated and categorized by region. Volume was compared to Global Burden of Disease (GBD) 2019 estimates for incidence of thermal injury by region. Journal submission instructions were reviewed for potential barriers to publication.

**Results:**

A total of 916 articles were reviewed from 59 countries. Analysis showed 147 articles with 1117 human photos. GBD incidence of burn, publications, and photographs by region are shown in Figure 1. North America, Europe, the Middle East, and Oceania had a higher proportion of published images to burn injury incidence than Latin America, Africa, and Asia (X2 (6, N = 915) =3585, p < 0.001). Barriers to journal publication included charges for published images (up to 600 USD/image), open access costs (900-3500 USD with 1 of 3 publishers offering tiered discounts specific to LMIC), English language review (0.06-0.07 USD/word), and society affiliation discounts. Table 1 summarizes identified barriers and potential solutions.

**Conclusions:**

There is a relative paucity of LMIC contributions to publications and clinical photos in English language burn journals. Identified publication barriers and added costs from journals likely contribute to this disparity.

**Applicability of Research to Practice:**

Publication-level strategies to increase authorship and clinical photographs from underrepresented regions are urgently needed.